# Air Pollution, Noise, Blue Space, and Green Space and Premature Mortality in Barcelona: A Mega Cohort

**DOI:** 10.3390/ijerph15112405

**Published:** 2018-10-30

**Authors:** Mark J. Nieuwenhuijsen, Mireia Gascon, David Martinez, Anna Ponjoan, Jordi Blanch, Maria del Mar Garcia-Gil, Rafel Ramos, Maria Foraster, Natalie Mueller, Ana Espinosa, Marta Cirach, Haneen Khreis, Payam Dadvand, Xavier Basagaña

**Affiliations:** 1 ISGlobal, 08003 Barcelona, Spain; mireia.gascon@isglobal.org (M.G.); mardav92@gmail.com (D.M.); maria.foraster@isglobal.org (M.F.); natalie.mueller@isglobal.org (N.M.); ana.espinosa@isglobal.org (A.E.); marta.cirach@isglobal.org (M.C.); H-Khreis@tti.tamu.edu (H.K.); payam.dadvand@isglobal.org (P.D.); xavier.basagana@isglobal.org (X.B.); 2 Universitat Pompeu Fabra (UPF), 08002 Barcelona, Spain; 3 CIBER Epidemiología y Salud Pública (CIBERESP), 28029 Madrid, Spain; 4 ISV Research Group, Research Unit in Primary Care, Institut Universitari d’Investigació en Atenció Primària Jordi Gol (IDIAP Jordi Gol), 17190 Catalonia, Spain; aponjoan@idiapjgol.info (A.P.); jblanch@idiapjgol.info (J.B.); mgarcia@idiapjgol.info (M.d.M.G.-G.); rramos.girona.ics@gencat.cat (R.R.); 5 Girona Biomedical Research Institute (IDIBGi), 17190 Catalonia, Spain; 6 Primary Care, Primary Care Services, Girona, Catalan Institute of Health (ICS), Catalonia, Spain; 7 Department of Medical Sciences, School of Medicine, University of Girona, 17004 Girona, Spain; 8 Center for Advancing Research in Transportation Emissions, Energy, and Health (CARTEEH), Texas A&M Transportation Institute (TTI), College Station, TX 77843, USA

**Keywords:** air pollution, green space, blue space, noise, mortality, city, cohort

## Abstract

*Introduction*: Cities often experience high air pollution and noise levels and lack of natural outdoor environments, which may be detrimental to health. The aim of this study was to evaluate the effects of air pollution, noise, and blue and green space on premature all-cause mortality in Barcelona using a mega cohort approach. *Methods*: Both men and women of 18 years and above registered on 1 January 2010 by the Sistema d’Informació pel Desenvolupament de la Investigació en Atenció Primària (SIDIAP) and living in the city of Barcelona were included in the cohort and followed up until 31 December 2014 or until death (*n* = 2,939,067 person years). The exposure assessment was conducted at the census tract level (*n* = 1061). We assigned exposure to long term ambient levels of nitrogen dioxides (NO_2_), nitrogen oxides (NO_x_), particulate matter with aerodynamic diameter less than 2.5 µm (PM_2.5_), between 2.5 µm and 10 µm (PM_2.5–10_, i.e., coarse particulate matter), less than 10 µm (PM_10_) and PM_2.5_ light absorption (hereafter referred to as PM_2.5_ absorbance) based on land use regressions models. Normalized Difference Vegetation Index (NDVI) was assigned based on remote sensing data, percentage green space and blue space were calculated based on land use maps and modelled road traffic noise was available through the strategic noise map for Barcelona. *Results*: In this large prospective study (*n* = 792,649) in an urban area, we found a decreased risk of all-cause mortality with an increase in green space measured as NDVI (hazard ratio (HR) = 0.92, 95% CI 0.89–0.97 per 0.1) and increased risks of mortality with an increase in exposure to blue space (HR = 1.04, 95% CI 1.01–1.06 per 1%), NO_2_ (HR = 1.01, 95% CI 1.00–1.02 per 5 ug/m^3^) but no risk with noise (HR = 1.00, 95% CI 0.98–1.02 per 5 dB(A)). The increased risks appeared to be more pronounced in the more deprived areas. Results for NDVI, and to a lesser extent NO_2_, remained most consistent after mutual adjustment for other exposures. The NDVI estimate was a little attenuated when NO_2_ was included in the model. The study had some limitations including e.g., the assessment of air pollution, noise, green space and socioeconomic status (SES) on census tract level rather than individual level and residual confounding. *Conclusion*: This large study provides new insights on the relationship between green and blue space, noise and air pollution and premature all-cause mortality.

## 1. Introduction

Urbanization is continuing at a rapid pace with more people than ever living in cities, which now accommodate over half the world’s population and almost 75% of the European population [1,2,3,4]. Cities often experience high levels of air pollution and noise and a lack of natural outdoor environments, which may be detrimental to health [5].

Long term exposure to traffic related air pollution [6,7] and noise [8] and the lack of green space [9] or blue space [10] exposure has been associated with premature all-cause mortality in independent studies. Until now, few studies have evaluated the risk of air pollution, noise and/or lack of green or blue space on premature all-cause mortality simultaneously, and evaluated whether the effects were independent.

Recently, a number of so called mega cohorts have started to evaluate relationships: between air pollution and mortality in the Netherlands [11], Italy [12], England [13], the US [14], and China [15]; between noise and mortality in the UK [8] and in Switzerland [16,17]; between blue space and mortality in Canada [18]; and between green space and mortality in Canada [18,19] and in Switzerland, the latter taking into account noise and air pollution as well [20]. These cohorts varied in size from 0.5 to 61 million subjects, which has the advantage of having high statistical power, but were at times limited in the accuracy of the exposure assessment and/or the number of potential confounders they could include. Further, only one study examined air pollution, green space and noise simultaneously [20].

The aim of this study was to evaluate the simultaneous effects of air pollution, road traffic noise and blue and green space on premature all-cause mortality in Barcelona using a mega cohort approach.

## 2. Methods

The city of Barcelona is located on the Spanish northeastern coast and capital of the Spanish autonomous community of Catalonia. As of 2012, Barcelona had 1,620,943 inhabitants living in an area of 101 km^2^ [21]. Barcelona has a Mediterranean climate with an annual mean temperature of 18 °C through mild winters and hot, humid summers [21]. Air pollution and noise levels are amongst the highest in Europe, due to Barcelona’s high population and traffic density, large share of diesel-powered vehicles, low precipitation, and an urban design of narrow street-canyons framed by semi-tall buildings of 5–6 stories. Green space is mainly located at the hilly west side of Barcelona and only 6.8 m^2^ is available per resident [21].

### 2.1. Study Population

Both men and women of 18 years and above living in Barcelona city and registered on 1 January 2010 by the Sistema d’Informació pel Desenvolupament de la Investigació en Atenció Primària (SIDIAP) were included in the cohort and followed up until 31 December 2014 or until death (*n* = 792,649 subjects, *n* = 2,939,067 person years). Most people in Spain use the public health care system. SIDIAP is a primary care computerized medical record of a representative sample of 5.8 million people (80% of the population) in Catalonia (Spain) [22,23]. Initial census tract location were used at the start of the study and updated when subjects moved residence within Barcelona. People moving outside Barcelona were censored at time of move.

The exposure assessment was conducted at the census tract level. There were 1061 census tracts in Barcelona with a median size of 3.6 hectares and average population of 1523.

### 2.2. Natural Space Assessment

We used two definitions of green space at the census tract level: (1) percentage green space within a census tract using land cover maps and (2) average greenness using satellite data. We added a 300 m buffer to the census tract estimates to account for surrounding greenness. The amount of green space within a census tract and the 300 m buffer was derived using Urban Atlas (2007, resolution 1:10,000) [24]. We estimated the percentage of green space within each census tract and the 300 m buffer. Average greenness of each census tract and surrounding 300 m buffer was determined as the average of the Normalized Difference Vegetation Index (NDVI). It was derived from the Landsat 8 at a spatial resolution of 30 m. NDVI is an indicator of greenness based on the difference between visible red and near-infrared surface reflectance. NDVI values range from −1 to +1, with higher values indicating more greenness. We obtained cloud-free images within the greenest season (April to July) during 2010–2014, the relevant years to our study. We used the NDVI data excluding big water bodies.

Also using Urban Atlas, we obtained information on water bodies and created a blue space indicator for the percentage of a water body (e.g., river, sea) in the census tract area.

### 2.3. Air Pollution Assessment

We assessed exposure to ambient levels of nitrogen dioxides (NO_2_), nitrogen oxides (NO_x_), particulate matter with aerodynamic diameter less than 2.5 µm (PM_2.5_), between 2.5 µm and 10 µm (PM_2.5–10_, i.e., coarse particulate matter), less than 10 µm (PM_10_) and PM_2.5_ light absorption (hereafter referred to as PM_2.5_ absorbance) for each year of the study. Our spatial assessment of exposure to air pollution was based on land use regression (LUR) models developed in the ESCAPE framework for Catalonia [25,26,27]. These models predicted 62–76% of variation in pollutant levels in our study area during 2008–2009. We created an artificial grid points data set with n random points within each census tract based on its area so increasing the density of points in smaller areas and reducing the number of points in larger areas. We ensured at least 5 observations predicted within each census areas. Air pollution was then averaged by census area. We assigned pollutant concentrations for each participant at the census tract level where they lived. Further details on this exposure assessment have been published elsewhere [25].

### 2.4. Noise Assessment

We applied the Strategic Noise Map of Barcelona [28] to estimate road traffic noise levels at the census tract level. The map was developed with a comprehensive set of standardized noise measurements, according to the Environmental Noise Directive 2002/49/EC (European Commission) [29]. Daytime traffic noise levels were calculated at the census-tract level using Barcelona’s strategic noise map (700–2300 h; LAeq 16 h) [28]. The LAeq 16 h was highly correlated (*r* > 0.95) with other noise measures including night and 24 h measures (Lnight, Lden). Noise maps were street-level maps so we overlaid it with the census tracts and we averaged exposure after a noise length weight procedure.

### 2.5. Mortality

The mortality data including date of death was extracted from the Sistema d’Informació pel Desenvolupament de la Investigació en Atenció Primària (SIDIAP) database. Only all-cause mortality was available with no information on the cause of death.

### 2.6. Covariate Data 

From the SIDIAP database, we extracted individual level covariates data for sex, age and smoking status. Besides, for each year of study at the census tract level, we obtained social economic status (SES). We used “Mortalidad en áreas pequeñas Españolas y Desigualdades socio-Económicas y Ambientales” MEDEA estimates on census tract level as the indicator for SES [30].

### 2.7. Linkage and Ethics Approval

Because of strict privacy reasons, the green and blue space, air pollution, noise data, covariate data, and mortality data were linked at the census tract level using the census tract area codes as recorded in the SIDIAP database. A variable was added for people who have moved during the follow up to easily identify movers. Ethics approval was obtained from the Institut d’Investigació en Atenció Primària Jordi Gol (IDIAP Jordi Gol) ethics committee.

### 2.8. Statistical Analyses

We used Cox proportional hazards regression models with time-dependent exposures and age as the time scale to estimate associations between exposure indicators and all-cause mortality. We reported hazard ratios (HRs). We adjusted for sex and smoking at an individual level and MEDEA at the census tract level. We conducted single and multiple exposure models. In addition, we evaluated potential effect modification by including an interaction term between blue space, green space, noise and air pollution indicators (one at the time) and the MEDEA (SES) score (above and below the median). Sensitivity analyses were conducted by removing completely those people that moved during follow-up and excluding people of 45 years and younger. We consider *p*-values < 0.05 as indication of statistical significance. All analyses were conducted in Stata14 software.

## 3. Results

Over the follow-up period (2010–2014), 28,391 deaths were recorded among the 792,649 study subjects (3.6%). Nearly 53% of the subjects were men and about 18% were smokers (Table 1). The average age at baseline was 50.9 years. Around 13% were in the most deprived category. In the 4-year follow-up period, 38,943 study subjects (4.9%) moved census tract areas. Greenness and percentage green space were generally low and average NO_2_ and PM_2.5_ levels were well above the WHO air pollution guideline values (Table 2). The correlation between the different exposures and some of the covariates was generally low to moderate, with some exceptions (Table 3). The highest correlation was between levels of PM_2.5_ absorbance and NO_2_ (*r* = 0.90).

After full adjustment, single exposure models showed a statistically significant decreased risk of mortality with increasing green space exposure as measured by NDVI (HR = 0.92 95% CI 0.89, 0.97), and statistically significant increased risks with NO_2_ (HR = 1.01 95% CI 1.00, 1.02) and blue space exposures (HR = 1.04 95% CI 1.01, 1.06) (Table 4). (The NDVI measure without a 300 buffer showed a less decreased risk (HR = 0.96 95% CI 0.94, 0.99)). There was no association with noise. Sensitivity analyses without movers did not substantially change the results, but for PM_2.5_ absorbance the magnitude of the hazard ratio increased and became statistically significant (HR = 1.03 95% CI 1.00, 1.06) (Table A1 in Appendix A). Excluding people 45 years or younger did not change the results.

Furthermore, there was little difference in the risk estimates for NDVI between the more and less deprived areas, but for percentage of green and blue space, PM_2.5_, PM_2.5_ absorbance and noise there were higher risks in the more deprived areas compared to the less deprived areas (Table 5).

Multiple exposure models with NDVI, PM_2.5_, PM_2.5_ absorbance, NO_2_ and noise in the same model (one air pollutant at the time) showed similar hazard ratios as for NDVI and noise in the single pollutant models, with some suggestion for a slight attenuation. But for PM_2.5_, PM_2.5_ absorbance and NO_2_ the hazard ratios were attenuated and/or became statistically non-significant (Table 6). The NDVI estimate was a little attenuated when NO_2_ was included in the model We did not run multiple exposure models with various air pollutants in the same model as the correlation among them was moderate to high (Table 3).

## 4. Discussion

In this large prospective study in an urban area we found a decreased risk of all-cause premature mortality with an increase in greenness measured as NDVI and increased risks of all-cause premature mortality with an increase in exposure to (1) blue space, measured as a percentage of the census tract area; and (2) air pollution, particularly NO_2_. The increased risks appeared to be more pronounced in the more deprived areas. Results for NDVI, and to a lesser extent NO_2_ remained most consistent after mutual adjustment for other exposures. The NDVI estimate was a little attenuated when NO_2_ was included in the model.

As with other mega cohorts [11,12,13,14,15,20], we found a relationship between air pollution and increased mortality with a similar magnitude compared to previous studies [11,12,13,14,15] confirming the already strong evidence base for air pollution and mortality [6]. As in the large mega cohort studies evaluating green space and mortality in Canada and Switzerland [18,19,20], we found a reduction in the risk of all-cause mortality with increasing NDVI. As in other studies, our exposure response relationship between green space exposure (NDVI) and reduced mortality remained after adjusting for air pollution, suggesting a modest, if any, mediatory role of air pollution in the association between the green space and mortality. In contrast to a previous study in Canada [10], where they found a decreased risk, we observed an increased risk in premature mortality with blue space. Also in contrast to an earlier study in London [8], we found no association between day-time noise and premature all-cause mortality.

We do not know why green space measured as NDVI reduced the risk of all-cause mortality while green space measured as percentage green space increased the risk for all-cause mortality, particularly in the more deprived areas, even though they were fairly highly correlated. It may be that the methods capture different types and sizes of green space with e.g., percentage green space only including green space above 0.5 hectares while NDVI includes all green. Vienneau et al. [20] on the other side found fairly similar risk reductions for their green space measures. Furthermore, we found an increased risk with the percentage blue space in the census tracts, particularly in the more deprived areas. However, less than a third of the participants were exposed to some percentage blue space. Perhaps blue space acted as a surrogate for the other exposures e.g., ship and ports related emissions and other chemicals, or we may not corrected sufficiently for confounders such as SES.

We did not see a difference in risk estimates for NDVI between the most and least deprived areas, as has been reported before, but we saw some substantially higher risk estimates for PM_2.5_, and PM_2.5_ absorbance in the more deprived areas, which may suggest that people living in these areas may be more vulnerable to these exposures. None of the other studies, except the UK and Canadian studies [13,19] looked at the risk distribution among the population. Similar to our study, Carey et al. [13] reported stronger associations between air pollution and mortality in the more deprived groups compared to the less deprived groups. Villeneuve et al. [19] reported stronger reduced risk estimates for green space in the more deprived groups compared to the less deprived groups.

### Strength and Limitations

The strength of the study is the large sample size and follow-up period, while the limitations are that some potential confounders are missing or were not available on individual level, specifically individual level SES. Important differences between neighborhood SES and individual level SES and exposure to air pollution have been observed before but were not consistent for different areas [31]. But the use of SES on census level may have led to some residual confounding and therefore not fully adjusting for SES. Furthermore, the mortality outcomes include accidental and natural cause mortality, as we did not have cause specific data. It is not obvious to what extent the accidental mortality may be affected by green space or blue space, but there is a possible mechanism through stress reduction/restoration leading to fewer accidental deaths. Unfortunately, it was not possible to separate accidental and natural cause mortality with the data available from SIDIAP. Furthermore, MEDEA 2001 data was used, and although there has a been little change in the spatial distribution of deprivation in Barcelona over the years, ideally census tract level SES data from 2011 would have been used but was not available. In addition, privacy concerns did not allow us to use household level geocodes. Therefore, our exposure assessment was at the census tract level and not at the household level, with the latter being more commonly used and being more accurate. This may have affected in particularly our noise estimates, but also possible other exposures, as noise tends to be more local and directional in effect. We expect this exposure misclassification to be non-differential, which would dilute the associations towards the null. Therefore, the magnitude of effects could potentially be stronger than observed. Finally, NDVI is a ‘catch all’ measure and it is not clear exactly what it means or consists of. However, we have already shown that NDVI is a good indicator for bushes, forest and particularly for urban green (as defined by the ecologic map of Barcelona) in Barcelona [32].

## 5. Conclusions

This large study provides new insights on the relationship between blue space, green space, noise and air pollution and all-cause mortality. Greenness (NDVI) showed a reduction in all-cause premature mortality while there was an increased risk of premature all-cause mortality with NO_2_ and blue space.

## Figures and Tables

**Table 1:** Characteristics of the population in Barcelona.

Characteristics	Barcelona Subjects *n* = 79,2649 (%)
Age at baseline; mean (SD)	50,9 (18,3)
Gender	
Males	416,943 (52,6)
Females	375,706 (47,4)
Smoking	
Non-smokers	595,328 (75,1)
Smokers	141,732 (17,9)
Ex-smokers	55,589 (7,0)
Social economic status (SES) (MEDEA)	
U1 Least deprived	266,907 (33,7)
U2	199,301 (25,1)
U3	124,029 (15,7)
U4	99,852 (12,6)
U5 Most deprived	102,560 (12,9)
Movers	
No	714,548 (90.2)
Yes	38,943 (4.9)
Missing	39,158 (4.9)

**Table 2:** Average exposure of the census tracts in Barcelona.

Exposures	GM (95% CI)	p25–p75
NDVI_census300 (2013, satellite data)	0.13 (0.13, 0.13)	0.12–0.15
Percentage of green spaces within census + 300 m buffer (%)	3.69 (3.67, 3.70)	0.00–6.00
Percentage of blue spaces within census + 300 m buffer (%)	0.31 (0.31, 0.31)	0.00–0.11
Mean annual concentration of NO_2_ (μg/m³)	53.42 (53.40, 53.45)	48.18–59.70
Mean annual concentration of PM_2.5_ (μg/m³)	16.08 (16.07, 16.08)	14.95–17.72
Mean annual concentration of PM_10_ (μg/m³)	38.29 (38.28, 38.30)	35.86–41.26
Mean annual concentration of PM_2.5_ Absorbance (BC) (10^−5^ m^−1^)	2.64 (2.64, 2.64)	2.37–2.91
Mean annual road traffic noise level (dB(A))	64.34 (64.53, 64.55)	62.21–67.28

GM = Geometric mean; CI = confidence interval; NDVI: Normalized Difference Vegetation Index.

**Table 3:** Correlations (Pearson) between different pollutants (residence during first year) and covariates in Barcelona. Particulate matter with aerodynamic diameter less than 2.5 µm (PM_2.5_), between 2.5 µm and 10 µm (PM_2.5–10_, i.e., coarse particulate matter), less than 10 µm (PM_10_) and PM_2.5_ light absorption (PM_2.5_ absorbance).

Exposure	% Green Space	% Blue Space	NO_2_	PM_2.5_	PM_10_	PM_2.5_ absorbance	Noise	NDVI	SES
**% Green Space**	1								
**% Blue Space**	0.12	1							
**NO_2_**	−0.59	−0.12	1						
**PM_2.5_**	−0.56	0.02	0.82	1					
**PM_10_**	−0.60	−0.04	0.82	0.77	1				
**PM_2.5_ absorbance**	−0.47	−0.06	0.90	0.87	0.77	1			
**Noise**	−0.24	−0.08	0.31	0.44	0.28	0.39	1		
**NDVI**	0.79	0.01	−0.57	−0.60	−0.50	−0.46	−0.25	1	
**SES**	0.28	0.13	−0.14	−0.29	−0.22	−0.17	0.19	0.19	1

**Table 4:** Models of single pollutants (PM_2.5_, NVDI, GS, BS, Noise) in Barcelona (*n* = 792,649).

Exposure	Model A		Model B		Model C	
	HR (95% CI)	*p*-Value ^1^	HR (95% CI)	*p*-Value ^1^	HR (95% CI)	*p*-Value ^1^
NDVI_census300 (2013, satellite data) (0.1 units)	0.97 (0.93,1.01)	0.130	0.91 (0.87,0.95)	0.000	0.92 (0.89,0.97)	0.001
% green spaces within census + 300 m buffer (10% increase)	1.03 (1.02,1.04)	0.000	1.01 (0.99,1.02)	0.470	1.01 (1.00,1.02)	0.170
% blue spaces within census + 300 m buffer (1% increase)	1.07 (1.04,1.09)	0.000	1.03 (1.01,1.06)	0.004	1.04 (1.01,1.06)	0.004
Mean annual concentration of NO_2_ (5 μg/m^3^)	1.01 (1.00,1.01)	0.010	1.01 (1.01,1.02)	0.000	1.01 (1.00,1.02)	0.001
Mean annual concentration of PM_2.5_ (5 μg/m^3^)	0.99 (0.96,1.02)	0.380	1.04 (1.01,1.07)	0.012	1.03 (0.99,1.06)	0.100
Mean annual concentration of PM_10_ (10 μg/m^3^)	0.97 (0.94,1.00)	0.090	1.01 (0.98,1.04)	0.510	1.00 (0.97,1.03)	0.790
Mean annual concentration of PM_2.5_ Absorbance (BC) (1 × 10^−5^ m^−1^)	1.01 (0.98,1.04)	0.480	1.03 (1.01,1.06)	0.017	1.02 (1.00,1.05)	0.089
Daily noise average level 2012 (5 dB(A))	0.97 (0.95,0.98)	0.000	1.00 (0.99,1.02)	0.670	1.00 (0.98,1.03)	0.820

^1^ Wald test; Model A: Adjusted for age and gender; Model B: Adjusted for age, gender and SES; Model C: Adjusted for age, gender, SES and smoking.

**Table 5:** Models of single pollutants (PM_2.5_, NVDI, GS, BS, Noise) by SES.

Exposure	MEDEA Index U1–U2 (*n* = 466,208) Least Deprived				MEDEA Index U3–U5 (*n* = 326,441) Most Deprived			
	Model D		Model E		Model D		Model E	
	HR (95% CI)	*p*-Value ^1^	HR (95% CI)	*p*-Value ^1^	HR (95% CI)	*p*-Value ^1^	HR (95% CI)	*p*-Value ^1^
NDVI_census300 (0.1 units)	0.92 (0.86,0.99)	0.017	0.93 (0.88,1.00)	0.054	0.92 (0.88,0.98)	0.008	0.94 (0.89,1.00)	0.038
% green spaces within census + 300 m buffer (10% increase)	1.01 (0.98,1.04)	0.570	1.01 (0.98,1.03)	0.370	1.02 (1.00,1.03)	0.048	1.02 (1.00,1.03)	0.016
% blue spaces within census + 300 m buffer (1% increase)	0.98 (0.92,1.04)	0.440	0.98 (0.92,1.04)	0.470	1.07 (1.04,1.09)	0.000	1.07 (1.04,1.09)	0.000
Mean annual concentration of NO_2_ (5 μg/m^3^)	1.01 (1.00,1.02)	0.120	1.01 (1.00,1.02)	0.220	1.02 (1.01,1.02)	0.000	1.01 (1.01,1.02)	0.001
Mean annual concentration of PM_2.5_ (5 μg/m^3^)	1.00 (0.95,1.04)	0.930	0.99 (0.95,1.03)	0.690	1.06 (1.02,1.11)	0.005	1.04 (1.00,1.09)	0.047
Mean annual concentration of PM_10_ (10 μg/m^3^)	0.96 (0.91,1.00)	0.076	0.95 (0.90,0.99)	0.024	1.04 (1.00,1.08)	0.066	1.02 (0.98,1.06)	0.290
Mean annual concentration of PM_2.5_ Absorbance (BC) (1 × 10^−5^ m^−1^)	1.00 (0.96,1.04)	0.990	1.00 (0.96,1.03)	0.870	1.07 (1.03,1.12)	0.000	1.06 (1.02,1.11)	0.005
Day time noise average level 2012 (5 dB(A))	1.00 (0.98,1.02)	0.950	1.00 (0.98,1.02)	0.900	0.99 (0.96,1.02)	0.500	0.99 (0.96,1.02)	0.420

^1^ Wald test; Model D: Adjusted for age and gender; Model E: Adjusted for age, gender and smoking.

**Table 6:** Multiple pollutant models (*n* = 792,649).

Pollutants	Model A		Model B		Model C	
	HR (95% CI)	*p*-Value ^1^	HR (95% CI)	*p*-Value ^1^	HR (95% CI)	*p*-Value ^1^
Model 1: PM_2.5_ and noise						
Mean annual concentration of PM_2.5_ (5 μg/m^3^)	1.02 (0.98,1.05)	0.310	1.04 (1.01,1.08)	0.012	1.03 (0.99,1.06)	0.098
Daily noise average level (5 dB(A))	0.96 (0.94,0.98)	0.000	1.00 (0.98,1.02)	0.660	1.00 (0.98,1.02)	0.740
Model 2: NO_2_ and noise						
Mean annual concentration of NO_2_ (5 μg/m^3^)	1.01 (1.01,1.02)	0.000	1.01 (1.01,1.02)	0.000	1.01 (1.00,1.02)	0.001
Daily noise average level (5 dB(A))	0.95 (0.94,0.97)	0.000	0.99 (0.97,1.01)	0.510	0.99 (0.97,1.01)	0.530
Model 3: NDVI, PM_2.5_ and noise						
NDVI_census300 (0.1 units)	0.93 (0.89,0.99)	0.012	0.91 (0.87,0.96)	0.001	0.92 (0.88,0.97)	0.002
Mean annual concentration of PM_2.5_ (5 μg/m^3^)	0.99 (0.95,1.03)	0.580	1.00 (0.96,1.04)	0.840	0.99 (0.95,1.03)	0.770
Daily noise average level (5 dB(A))	0.96 (0.94,0.98)	0.000	1.00 (0.98,1.02)	0.700	1.00 (0.98,1.02)	0.770
Model 4: NDVI, NO_2_ and noise						
NDVI_census300 (0.1 units)	0.99 (0.93,1.04)	0.570	0.94 (0.89,0.99)	0.016	0.95 (0.90,1.00)	0,046
Mean annual concentration of NO_2_ (5 μg/m^3^)	1.01 (1.00,1.02)	0.001	1.01 (1.00,1.02)	0.030	1.01 (1.00,1.01)	0,087
Daytime noise average level (5 dB(A))	0.95 (0.94,0.97)	0.000	0.99 (0.97,1.01)	0.450	0.99 (0.97,1.01)	0,470
Model 5: NDVI and noise						
NDVI_census300 (0.1 units)	0.94 (0.90,0.98)	0.008	0.91 (0.87,0.95)	0.000	0.92 (0.89,0.97)	0.001
Daytime noise average level (5 dB(A))	0.96 (0.94,0.98)	0.000	1.00 (0.98,1.02)	0.730	1.00 (0.98,1.02)	0.700
Model 6: NDVI and NO_2_						
NDVI_census300 (0.1 units)	1.00 (0.95,1.05)	0.930	0.94 (0.89,0.99)	0.017	0.95 (0.90,1.00)	0.050
Mean annual concentration of NO_2_ (5 μg/m^3^)	1.01 (1.00,1.02)	0.039	1.01 (1.00,1.02)	0.039	1.01 (1.00,1.01)	0.110
Model 7: NDVI and PM_2.5_						
NDVI_census300 (0.1 units)	0.93 (0.89,0.98)	0.011	0.91 (0.87,0.96)	0.001	0.92 (0.88,0.97)	0.002
Mean annual concentration of PM_2.5_ (5 μg/m^3^)	0.96 (0.92,1.00)	0.027	1.00 (0.96,1.04)	0.930	0.99 (0.95,1.03)	0.700
Model 8: PM_2.5_ absorbance and noise						
Mean annual concentration of PM_2.5_ absorbance (BC) (1 × 10^−5^ m^−1^)	1.04 (1.01,1.07)	0.010	1.03 (0.99,1.06)	0.098	1.03 (1.00,1.06)	0.085
Daytime noise average level (5 dB(A))	0.96 (0.94,0.97)	0.000	1.00 (0.98,1.02)	0.740	1.00 (0.98,1.02)	0.720
Model 9: NDVI, PM_2.5_ absorbance and noise						
NDVI_census300 (0.1 units)	0.96 (0.91,1.00)	0.071	1.01 (1.00,1.02)	0.001	0.93 (0.89,0.97)	0.003
Mean annual concentration of PM_2.5_ absorbance (BC) (1 × 10^−5^ m^−1^)	1.03 (0.99,1.06)	0.099	0.99 (0.97,1.01)	0.530	1.01 (0.97,1.04)	0.710
Daytime noise average level (5 dB(A))	0.96 (0.94,0.97)	0.000	0.92 (0.88,0.97)	0.002	1.00 (0.97,1.02)	0.630
Model 10: NDVI and PM_2.5_ absorbance						
NDVI_census300 (0.1 units)	0.97 (0.92,1.01)	0.170	0.99 (0.95,1.03)	0.770	0.93 (0.89,0.97)	0.003
Mean annual concentration of PM_2.5_ absorbance (BC) (1 × 10^−5^ m^−1^)	1.00 (0.97,1.03)	0.990	1.00 (0.98,1.02)	0.770	1.00 (0.97,1.03)	0.810

^1^ Wald test; Model A: Adjusted for age and gender; Model B: Adjusted for age, gender, and SES; Model C: Adjusted for age, gender, SES, and smoking.

## Appendix A

**Table A1:** Models of single pollutants (PM_2.5_, NVDI, GS, BS, Noise). Excluding movers. *n* = 753,706.

Exposure	Model A		Model B		Model C	
	HR (95% CI)	*p*-Value ^1^	HR (95% CI)	*p*-Value ^1^	HR (95% CI)	*p*-Value ^1^
NDVI_census300 (2013, satellite data) (0.1 units)	0.96 (0.92,1.00)	0.058	0.90 (0.87,0.94)	0.000	0.92 (0.88,0.96)	0.000
% green spaces within census + 300 m buffer (10% increase)	1.03 (1.01,1.04)	0.000	1.00 (0.99,1.02)	0.560	1.01 (0.99,1.02)	0.210
% blue spaces within census + 300 m buffer (1% increase)	1.06 (1.04,1.09)	0.000	1.03 (1.01,1.06)	0.006	1.03 (1.01,1.06)	0.006
Mean annual concentration of NO_2_ (5 μg/m^3^)	1.01 (1.00,1.02)	0.002	1.01 (1.01,1.02)	0.000	1.01 (1.01,1.02)	0.000
Mean annual concentration of PM_2.5_ (5 μg/m^3^)	0.99 (0.96,1.02)	0.610	1.04 (1.01,1.08)	0.005	1.03 (1.00,1.06)	0.054
Mean annual concentration of PM_10_ (10 μg/m^3^)	0.98 (0.95,1.01)	0.150	1.01 (0.98,1.04)	0.360	1.00 (0.97,1.03)	0.990
Mean annual concentration of PM_2.5_ Absorbance (BC) (1 × 10^−5^ m^−1^)	1.02 (0.99,1.04)	0.260	1.04 (1.01,1.07)	0.006	1.03 (1.00,1.06)	0.039
Daily noise average level 2012 (5 dB(A))	0.97 (0.95,0.99)	0.000	1.0 (0.99,1.03)	0.530	1.00 (0.99,1.02)	0.660

^1^ Wald test; Model A: Adjusted for age and gender; Model B: Adjusted for age, gender and SES; Model C: Adjusted for age, gender, SES and smoking.
